# Dr. Hood on the External Application of Nitrate of Silver in Various Diseases

**Published:** 1829-07-01

**Authors:** 


### XXXI.

### On the External Application of Lunar Caustic in Various Diseases. By S. Hood, M.D.

[Analytical Physiology, 2d. Edit.]

Soon after our review of Mr. Higginbottom's work appeared, in No. XX of this Journal, we received a somewhat remonstrative letter from Dr. Hood, in which he claimed the merit of priority, as to the use of lunar caustic, over the author just named, and complained that we had done him a literary injustice, not only by passing his claim over unnoticed, but by giving Mr. H. the credit of originality in the application of the remedy. Not knowing where Dr. Hood's observations were to be found, we wrote to that gentleman, and were referred to his analytical physiology, the first edition of which was published in June, 1822—consequently before the first edition of Mr. Higginbottom's work saw the light. That neither of the authors ever saw the first edition of the other's book, we firmly believe,—and it is hardly necessary to say, that we were entirely unacquainted with the claims of Dr. Hood, till we turned to the analytical physiology, in which we should never have dreamt of finding practical observations on the external use of nitrate of silver. We shall do Dr. Hood every justice by noticing those parts of his work in which the remedy is alluded to.

At page 14, the following observations occur.

"In patients afflicted with hemiplegia, the circulation is in general languid on the whole of the paralytic side; but even in all such cases it is easy to raise its temperature to an equality with that of the whole side, by a lunar caustic eschar, if the apoplectic symptoms have for some time disappeared. A case of this description I shall relate under the head of Muscular Irritability. As an eschar of lunar caustic, over the nerve of a paralytic limb, causes an augmentation of heat through all its ramifications, it necessarily follows, that the nervous system must contribute greatly to the generation of animal heat; but as the nervous system may be made to cease its calorific action, by the abstraction of blood, animal heat must therefore be a product arising from the combined action of the blood and nervous system, and does not depend solely upon either the one or the other."

Again at page 32 we have a case connected with the present subject.

"April 6th, 1819.—Hugh Ward, private dragoon of the army of Bengal, aged thirty-six years, joined a detachment of the 25th cavalry, on board the Mangles transport, at Madras, bound for England. He was affected with hemiplegia of the right side. His arm was shrunk and deprived of all voluntary motion; the flexor muscles of the fingers and the extensor longus pollicis were contracted, the fingers were in consequence closed, and the thumb drawn radiad; the leg and thigh were shrivelled, but he could walk a little. His disease had commenced by a stroke of apoplexy, three months previous, during which period he had remained in the Garrison Hospital of Madras, where he had been repeatedly bled and blistered.

"April 11th.—An eschar was made in the right axilla with the nitrate of silver. On the following morning, when questioned respecting the effect of the eschar, he replied, 'Shortly after the issue was made, I felt my hand open.' This being the first paralytic patient that I had treated with lunar caustic, I was not a little surprised to see him move his fingers, wrist, and fore-arm with facility. The pulsation of the radial artery was become much stronger; and a thermometer placed in either hand stood at 100°, the temperature of the atmosphere between decks being 90°.

"It became shortly after evident, that in future, it would be necessary to make the eschar over the cervical vertebræ, for the muscles of the shoulder-joint, notwithstanding the intimate connexion of its nerves with the axillary plexus, continued quite motionless.

"April 25th.—A new eschar was made over the third cervical vertebra, with the view of influencing the nervous action of both extremities by one application. The effect was nearly as remarkable as that of the first eschar; for, two hours after its application, he could move the humerus (which till now had been motionless) backwards and forwards, or hold it at a right angle with his body. The pulsation was not so strong when the eschar was in the axilla, but the temperature of the hand was still at 100°. In order to exercise his muscles, he was directed to lift weights and increase them as he acquired strength; in six weeks, the eschar being renewed at intervals of ten or twelve days, he could carry sixty pounds, and write his own name.

"The cure was thus advancing slowly and successfully, but it did not keep pace with my wishes; the eschar was renewed on the back of the neck, and a fresh one made over the first lumbar vertebra. Instantly after the second application, the right arm felt numb, and the patient could not close his hand, owing, apparently, to the excessive expansion of the muscles; the pulse was strong, head-ache troublesome, and the animal heat increased. In the course of two days, he took three doses of antimonial wine; all the disagreeable symptoms disappeared, and the cure went on as usual.

"After the sloughs fell off, I tried to carry on the cure by frictions with the liniment of ammonia, but the arm remained quite stationary; cold affusions of water on the paralytic side, and preparations of cinchona had no better effect. After losing a whole month in these fruitless expedients, I resumed the application of eschars. The remainder of this case is too tedious to relate here; it is enough to state, that before the patient disembarked at Greenwich, October 21st, he could carry two hundred pounds' weight and walk pretty well, considering that he had had so little opportunity of exercising his limbs.

"The circumstance to which I would particularly draw the attention of the reader in this case, is the expansion of the flexor muscles of the wrist and fingers after the first eschar; the involuntary opening of the hand was the first effect from the augmentation of the vital force by the lunar caustic, which was too obvious to escape even the patient's observation."

We find no more notice of the nitrate of silver, in the volume before us, till we come to the subject of Neuralgia, at page 101 of the work. Dr. Hood appears to entertain some peculiar opinions respecting this dire disease, and which we do not profess to clearly understand. Neuralgia, in his experience, has always resulted from the application of cold. "In neuralgia," he observes, "there is a diminution of arterial, as well as of venal expansibility; but the delicate veins of the sciatic neurilema being most easily affected, they collapse and are unable to absorb the blood from their capillaries, which become distended, causing intolerable pain of the sciatic nerve beyond the ankle, where the points of contact between the blood-vessels and divisions of this nerve become multiplied." So much for Dr. Hood's theory of the disease. We shall quote his own words as to the treatment.

"When the expansibility of the veins of the sciatic neurilema is renewed, by restoring the action of the nerve with the nitrate of silver, the disease is removed, by absorption, in a few days. But if the application of the lunar caustic be so great as to affect the expansibility of both the arteries and veins, the pain is more severe for a day or two; but on the fifth or sixth day, the expansibility of the arteries sinks to its natural state, while that of the veins continues preternaturally augmented till the tenth; before which period, the capillaries are usually restored to their natural state by venal absorption. It is precisely upon this principle, of keeping up a corresponding degree of expansibility between the arteries and veins, that stimulants are beneficial in scalds.

"Dr. Sillar, who has had extensive practice in neuralgia of the sacral extremities, informs me, that it is his opinion, if the lunar caustic could be applied in such a manner as to raise the expansibility of the veins to its healthy pitch, without influencing that of the arteries, the relief would generally be almost immediate. I feel much disposed to adopt this opinion; in favour of which, he possesses several beautiful cases in his Journal.

"The paralytic stage of neuralgia being merely a temporary diminution of the vital force, the patient often feels himself more than half cured when the practitioner has finished the eschar. It is of great consequence to cure neuralgia of the sacral extremities during the first three months of the inflammatory stage; for when it has lasted more than a year, the patient is very obnoxious to relapse."

White swelling is another disease to the cure of which Dr. Hood has directed the nitrate of silver. He thinks it will hardly be denied that "white swelling of the knee-joint proceeds from defective lymphatic absorption — or, in other words, morbid contraction of the lymphatic tubes." He endeavours to place this in a clear light by the following experiment.

"When a lunar caustic eschar is applied to a white swelling of the knee-joint, the expansibility of the absorbents is renewed; but although there be generally no inflammation except at the edge of the slough, yet the extreme arteries of the joint are also a little expanded the first three or four days. But by the twelfth day of the eschar, the swelling has subsided sometimes a full inch by absorption alone; for lunar caustic causes scarcely any discharge of pus. The sudden abstraction of the effused fluid, in a diseased joint thus treated, cannot be supposed to result from the lymphatics alone; the veins, doubtless, contribute material assistance: but the principle of venal and lymphatic absorption is the same."

In an appendix, entitled "Practical Observations," Dr. Hood has again adverted to the external use of nitrate of silver in the cold stage of ague, which he considers to proceed from a diminution of the vital force.

"It was," says he, "from a deep impression of the truth of this conclusion that I recommended Dr. Sillar to apply externally the nitrate of silver in a case of Quartan Ague, which had lasted upwards of twelve months, and had resisted both bark and arsenic. The eschar was made an hour before the accession of the paroxysm; we returned to see the patient at the period when the cold stage usually ended, but it had not come on; our entrance roused him from a comfortable sleep. It would be superfluous to descant here on this synthetic proof of Cullen's theory; I leave it for the discussion of the contending theorists of the present day."

The last passage which we shall extract from Dr. Hood's work, and which, indeed, is the only other one which we could find on the subject of lunar caustic, will be found between pages 195 and 199, of the volume.

"Hemiplegia.—In these kingdoms, the treatment of acute hemiplegia cannot be improved. Bleeding and blistering often remove this disease in the course of six weeks or two months; but if, at the end of this period, the use of the paralytic side be not partially restored, a cure is generally considered hopeless. If, however, all the apoplectic symptoms be entirely removed, and there remain no appearance of plethora of the cerebral blood-vessels, even chronic hemiplegia may sometimes be greatly benefited, by a succession of small lunar caustic eschars. It is impossible to say, *à priori*, in what case this treatment may be successful; but this information is very easily ascertained, by making an eschar over the course of the nerves of the paralytic arm, either in the axilla, or above the clavicle; and if either the sense or motion of the arm be improved, just expectation may be entertained of alleviating the disease.

"The nervous system of those individuals who possess the sanguine temperament, is most easily excited by the nitrate of silver; a circumstance which is to be borne in mind, when this substance is applied to the spine. But when hemiplegia is relieved by lunar caustic, the cure goes on as fast when the eschar is about half the size of a sixpence, as when it is much larger; the nervous system requires a constant, but mild stimulant. The applications may be made on the back of the neck, and renewed every twelve or fourteen days. The longer a patient has been under the operation of the nitrate of silver, the more sensible he becomes to its influence; but if the treatment be pursued in a judicious manner, and only in proper cases, no untoward circumstances can arise from its use.

"NEURALGIA.—The eschars for the cure of neuralgia of the sacral extremities, may vary from the size of a shilling to that of half-a-crown. The caustic must be applied over the course of the affected nerve, and rubbed till the part assumes a red colour studded with grey spots. Rest and warmth are to be strictly enforced during the cure, and a considerable time after recovery."

"The cure of white-swelling should be commenced by the application of a dozen or two of leeches to the joint, unless the patient's general health be already too weak, to bear even local bleeding. Leeches are preferable to cupping, as they do less mechanical injury to the joint. Two days after the bleeding, a lunar caustic eschar, as large as half-a-crown, must be made at one side of the patella, and the whole joint must be enveloped in a poultice, and maintained perseveringly in a state of rest. When the eschar comes away, the poultice may be discontinued, and the sore dressed with cerate or dry lint, and allowed to heal without delay. When the sore is quite skinned over, a fresh eschar may be made on the other side of the patella, and treated in every respect like the first. It is not amiss to keep a measure of the joint, to see the progress of the cure. For constitutional treatment, I generally employ alterative doses of calomel and James's powder; but I am not certain that much good is derived from these medicines. Small doses of rhubarb are sometimes beneficial. When sinuses exist, they must not be laid open, if the cure be attempted on the principle of absorption. The patient should not be allowed to walk about for some weeks after the swelling is gone, and cold must be carefully avoided. By this treatment, the patient suffers comparatively little, and the cure is almost certain, if the constitution be otherwise good, and the disease recent. The actual cautery, blisters, and potass eschars, frequently defeat the purpose of the practitioner, from the violence of their operation. As white-swelling occurs most commonly in scrofulous constitutions, it is consequently often combined with diseases of the other organs, such as tabes mesenterica, tubercles of the lungs, rickets, and hydrocephalus; it is not, therefore, to be presumed, that by any local application, the scrofulous tendency of the animal economy can be eradicated.

"Many facts which I already possess, as well as rigorous analogy, might induce me to recommend the external application of lunar caustic in some other obstinate diseases; but I am unwilling to promulgate any opinion, of the accuracy of which my own mind is not entirely satisfied; and I venture to assert, that it presents the physiological practitioner with curative means, for which the *res medica* offers no substitute. I should even here have enlarged more on its medical properties, were it not that my friend Dr. Sillar is now occupied on this subject, and will perhaps lay his observations before the public."

We hope we have now done Dr. Hood ample justice, as to his claims of priority in the external use of lunar caustic. How far his observations interfere with or anticipate those of Mr. Higginbottom, we shall not undertake to decide. The authors were evidently unacquainted with each other's writings—and so far they tend to strengthen one another, without any necessity for a war as to priority. We shall be anxious to see the announced publication of Dr. Sillar, since we have reason to believe that the range of diseases to which the nitrate of silver may be applicable, is not yet defined. We hope, indeed, ere long, to be able to communicate to our professional brethren some very important cases where the internal use of the nitrate of silver was followed by the most beneficial effects—cases, too, in which the remedy has never before been tried.

